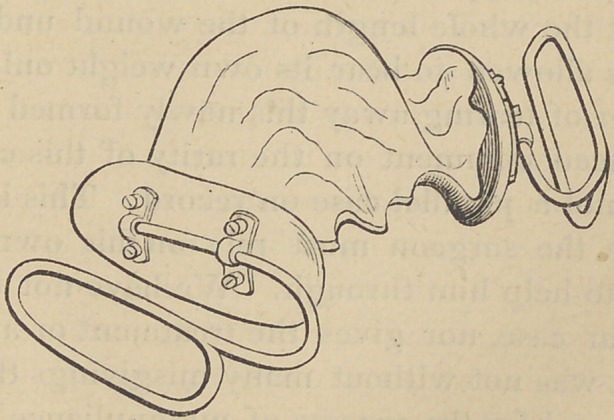# Fracture of the Facial Bones

**Published:** 1876-09

**Authors:** S. B. Dewey

**Affiliations:** Cleveland, O.


### FRACTURE OF THE FACIAL BONES.

S. B. DEWEY, CLEVELAND, O.

June 2d, F. S., aged twenty. A loaded wagon passed over his face from left to right, striking the external angular process of the frontal bone first, passing across the left orbit, crushing the eye out, and passing upward to the frontal bone at the root of the nose, cutting the nose free from the skull, breaking the bridge and fracturing the turbinated bones. From this point it glanced off from the frontal, taking a downward and outward direction, passing beneath the right eye, crushing the facial portion of the superior maxillary through into the antrum, forcing the right malar bone upward, and outward, fracturing the roof of the mouth and forcing a spicula of bone through its mucous surface, thus making an external wound between two and one half and three and one-half inches in length, cutting through the soft parts, and making an opening through which you could look into the throat and meatus of the nose, and allowing the face to drop down.

Dr. C. E. Buell, of East Cleveland, was called to dress the wound and place the parts in position. After cleansing it and removing the loose fragments of bone, the parts were brought together and retained by stitches and adhesive strips. The vessels being so crushed that it was impossible to take them up; the hæmorrhage was not alarming. In two or three days the stitches pulled out letting the whole superior maxillary fall down, and so remaining until the 15th inst. On the 11th, Dr. Buell (at the suggestion of Dr. H. F. Biggar) called to know if I could do anything for his case, he having failed to keep the parts in position. On the 13th, in company with Dr. B., I visited his patient with the intention of making a thorough examination of the case and render such assistance as might be deemed advisable.

On making a careful examination (this was eleven days after the accident), I found the face had fallen down so that a gap one and one-half inches wide was left between the frontal bone and the severed end of the nose. The edges of the wound granulating, the discharge of pus great in quantity, but mostly healthy. An impression was now taken with wax of the roof of the mouth, alveolar arch and teeth; which was attended with considerable difficulty, the parts being so mobile that it was almost impossible to put pressure enough on it to make an impression in the softened wax. After the impression was obtained we were again troubled to withdraw it without displacing the wax.

From this impression a plaster cast was obtained, over which a vulcanite plate was made to cover the roof of the mouth and alveolar ridge. The labial surface of this plate, over the four incisors was cut away, giving a free passage into the mouth, through which nourishment could be taken.

On both buccal surfaces of the plate were attached two silver wires, each six inches in length and one-twelfth an inch in diameter. The plate end of each wire was armed with two braces an inch apart, each brace having two screws passing through it into the plate. The first brace being attached between the canine and first bicuspid teeth, the second opposite the first molar. These wires passed out at the corners of the mouth and were bent directly back along the side of the cheek being parallel with the sides of the plate in the mouth, for about two inches, and then curved downward forming a loop through which a bandage could be passed and fastened to a skull cap above.

APPARATUS USED BY DR. DEWEY

The 15th inst. this appliance was adjusted and the gap closed about one third. The wound having been agap so long before the splint was applied, and the discharge of pus so great, that it appeared advisable to close up the opening gradually.

My next visit was made on the 17th. Found some improvement in him since the application of the splint. This time the gap was closed about one third of an inch more; also some spicula of bone removed. The right eye was very much inflamed from sympathetic connection with the dead one on the left side, which was now nothing but a sack of pus.

It was considered best to remove the left eye for the benefit of the right. This was done on the 19th by Dr. W. A. Phillips, after the administration of an anæsthetic.

The 21st I saw the patient again; was doing well, still a little weak from the effects of the anæsthetic. The gap was now closed to within three-eighths of an inch; a slight union had taken place under the right eye.

It was about ten days (July 2d) before I saw the patient again. The improvement during this time was very marked, He had been around the house and out doors for two days. The gap was almost entirely closed up, only a slight opening over the bridge of the nose. The opening into the throat was also closed, so that the injections used to cleanse the parts no longer passed into it. On removing the plate we found the parts were self-sustaining, union having taken place almost the whole length of the wound under the right eye. It was allowed to bear its own weight only for a moment for fear of tearing away this newly formed tissue.

I hardly need comment on the rarity of this case. There probably is not a parallel case on record. This is one of the cases where the surgeon must rely on his own immediate production to help him through. We have not a book that cites a similar case, nor gives the treatment or appliances to be used. It was not without many misgivings that this was undertaken, and for the success of my appliance I am greatly indebted to my colleague Dr. D. R. Jennings for his suggestions and advice given during its construction.

Everything appeared to be in our favor in this case. We had a young, healthy man, whose manner of living was plain and substantial, with a constitution strong enough to withstand almost any strain. Nor was this strength allowed to be wasted but under Dr. Buell's skillful treatment it was kept up surprisingly well. And again Dr. B. is entitled to much credit for the thorough manner in which he kept this wound cleansed and disinfected. As to the constitutional treatment and dressings followed in the case, I am not sufficiently conversant to give it in detail, (the writer having only been present at the stated periods to cleanse and replace the splint) but as far as my knowledge goes. it was thorough and scientific, being creditable both to himself and his profession.

In glancing over this report it will be seen that from the day the splint was put in place, the patient began to mend; in less than six days we had the gap closed within three-eighths of an inch, the discharge of pus growing less and more healthy. Before its application the doctor had well grounded fears of pyæmia (of which he spoke on my first visit) which gradually subsided as soon as the parts were supported. At the time of writing the patient is rapidly improving. All that remains to be done is to wait for nature to do her work, her promises being very flattering for a fair appearing face in the course of a few more days.

## Figures and Tables

**Figure f1:**